# Circ_0006332 promotes growth and progression of bladder cancer by modulating MYBL2 expression via miR-143

**DOI:** 10.18632/aging.102481

**Published:** 2019-11-22

**Authors:** Mingshan Li, Yili Liu, Jie Liu, Wei Li, Ning Li, Dongwei Xue, Xiling Zhang, Ping Wang

**Affiliations:** 1 Fourth Affiliated Hospital of China Medical University, Shenyang 110032, China; 2 Science Experiment Center of China Medical University, Shenyang 110122, China

**Keywords:** circ_0006332, MYBL2, bladder cancer, proliferation, invasion

## Abstract

In this study, we analyzed the role of circular RNAs in the growth and progression of bladder cancer. Direct Sanger sequencing and quantitative RT-PCR analysis showed that circ_0006332 was significantly upregulated in bladder cancer tissues. Sequencing analysis showed that circ_0006332 is generated from splicing of exons 8 and 9 of the *MYBL2* transcript. Fluorescence *in situ* hybridization analysis showed that circ_0006332 was localized to the cytoplasm of bladder cancer cells. Dual luciferase reporter assays showed that miR-143 specifically bound to circ_0006332 and the 3’UTR of MYBL2. High expression of circ_006332 correlated with tumor-node-metastasis stages and muscular invasion in bladder cancer patients. Knockdown of circ_0006332 in bladder cancer cells decreased proliferation, colony formation and invasiveness. Circ_0006332 knockdown increased E-cadherin levels and decreased Vimentin, CCNB1 and P21 protein expression. This suggests that circ_0006332 promotes epithelial–mesenchymal transition and cell cycle progression. *In vivo* experiments in nude mice showed that circ_0006332 knockdown bladder cancer cells form significantly smaller tumors than the controls. Our study demonstrates that circ_0006332 promotes the growth and progression of bladder cancer by modulating MYBL2 expression by acting as a sponge for miR-143. Circ_0006332 is thus a potential early diagnostic marker of bladder cancer.

## INTRODUCTION

Bladder cancer is one of the most prevalent urinary tumors with about 550,000 new cases and 200,000 deaths reported worldwide in 2018 [1]. Several genetic and environmental factors are associated with bladder cancer risk [2]. Surgery is the main treatment for bladder cancer patients. The 5-year survival rate for bladder cancer patients is low because of high risk of recurrence [3, 4]. Moreover, the genetic mechanisms that promote initiation, growth, and progression of bladder cancer are not well understood. Therefore, there is an urgent need to identify new biomarkers for early diagnosis of bladder cancer.

In recent years, several studies have discovered that noncoding RNAs play a significant role in tumorigenesis and cancer progression [5–7]. One category of noncoding RNAs are circular RNA (circRNA), which are characterized by a closed-loop structure without a 5′ cap and a 3′ tail. A well-defined function of the circRNAs is to regulate protein-miRNA interactions by acting as a sponge for specific microRNAs (miRNAs) [8]. CircRNAs participate in both physiological and pathological processes, including growth and progression of malignant neoplasms [9, 10]. The circRNA transcripts of *PGM5* and *KIAA1462* genes are significantly downregulated in bladder cancer [11]. Low expression of circ_00018069, a circRNA transcript from the *KIAA1462* gene, is associated with the differentiation and muscular invasion of bladder cancer by modulating ErbB, Ras, FoxO and focal adhesion signaling pathways [12]. In the present study, we analyzed RNA-seq data and identified several differentially expressed circRNAs in bladder cancer tissue samples. The circRNA transcripts of the MYB Proto-Oncogene Like 2 (*MYBL2*) and Cyclin B1 (*CCNB1*) genes were significantly upregulated in the bladder cancer tissue samples. This included circ_0006332, which is generated by splicing from the *MYBL2* gene. We demonstrated that circ_0006332 increases MYBL2 levels in bladder cancer tissues and cell lines by sponging miRNA-143. Knockdown of circ_0006332 decreased bladder cancer cell proliferation, colony formation and invasiveness. *In vivo* xenograft experiments in nude mice showed that bladder cancer cells with circ_0006332 knockdown form significantly smaller tumors compared with the controls. Overall, our data suggests that circ_0006332 increases MYBL2 protein levels by sponging miR-143 in bladder cancer tissues and cell lines. We postulate that circ_0006332 is a potential early diagnostic biomarker of bladder cancer.

## RESULTS

### Circ_0006332 is differentially expressed in bladder cancer tissues

**Figure 1:**
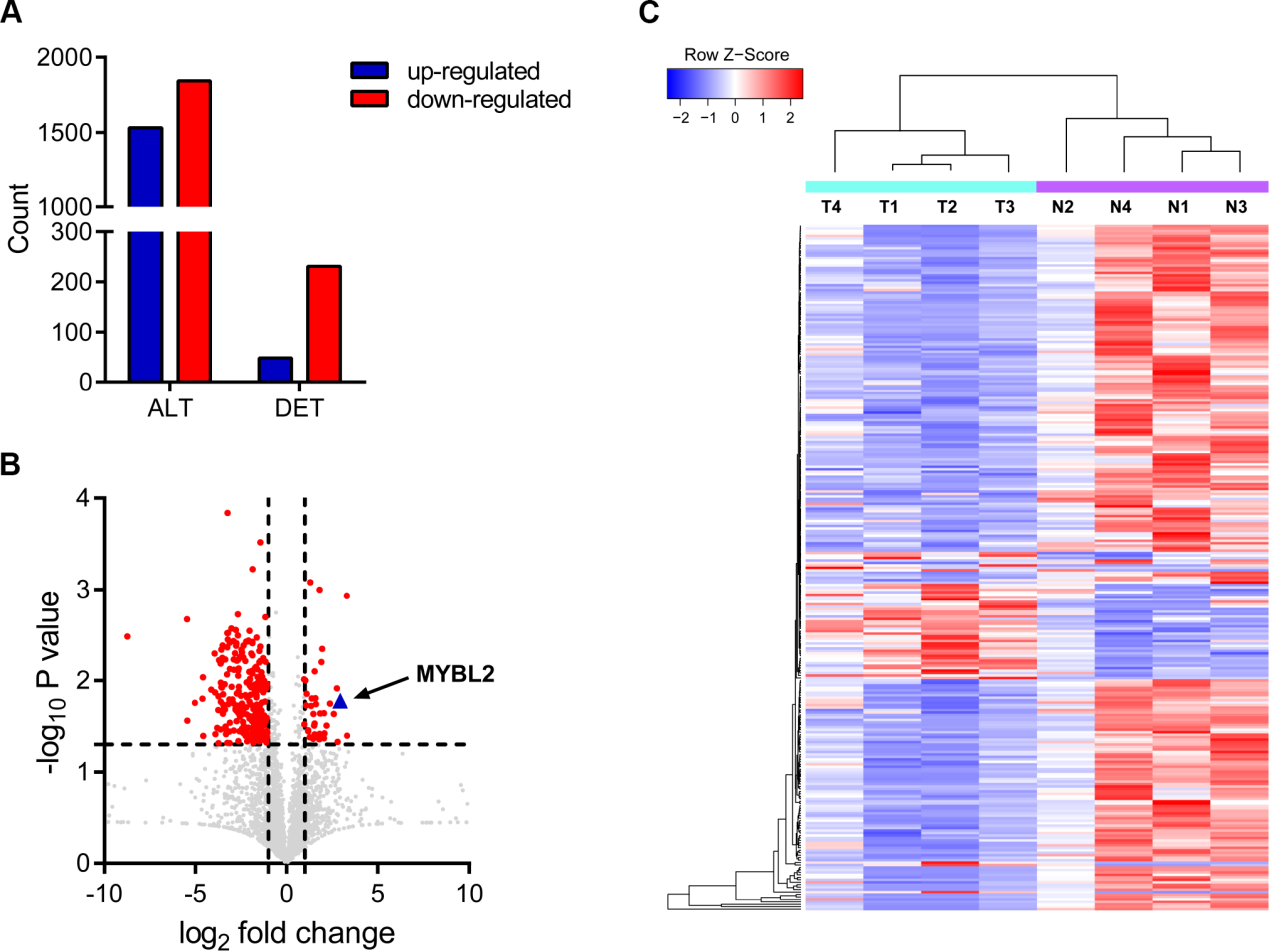
**Circular RNA expression profiles in bladder cancer and adjacent normal tissues.** (**A**) The chart shows all detected (ALT) and differentially expressed (DET) circRNA transcripts from the RNA-seq analysis. (**B**) Volcanic plot of circRNA transcripts. The vertical lines correspond to 2-fold increase (upregulation) or decrease (downregulation) in circRNA expression. The horizontal line corresponds to P = 0.05. The red points correspond to circRNA transcripts with a fold-change > 2.0 and P < 0.05. As shown, a 7.94 fold upregulation of circRNA transcripts of MYBL2 is observed in the bladder cancer tissues. (**C**) The clustering diagram shows 48 upregulated and 231 downregulated circRNA transcripts in bladder cancer tissues (T) compared with the adjacent normal bladder tissues (N).

We identified 3377 circRNA transcripts by whole transcriptome sequencing analysis, including 1340 upregulated and 1844 downregulated circRNAs. Among these, 279 circRNA transcripts were differentially expressed including 48 upregulated and 231 downregulated transcripts (Figure 1A–1B). Hierarchical clustering analysis showed distinct circRNA expression patterns between cancerous and adjacent normal tissues (Figure 1C). We selected top ten dysregulated circRNA transcripts, including 5 upregulated and 5 downregulated transcripts for further analysis. The circRNA IDs are listed in Supplementary Table 1. Circ_0087138, circ_00018069, circ_0006332 and circ_0001495 were significantly dysregulated in bladder cancer tissues. Sequencing analysis showed that circ_0006332 is generated by splicing within the *MYBL2* transcript and significantly upregulated in bladder cancer tissues (Figure 1C). Therefore, we selected circ_0006332 for further study.

### Basic characteristics and clinical significance of circ_0006332

**Figure 2:**
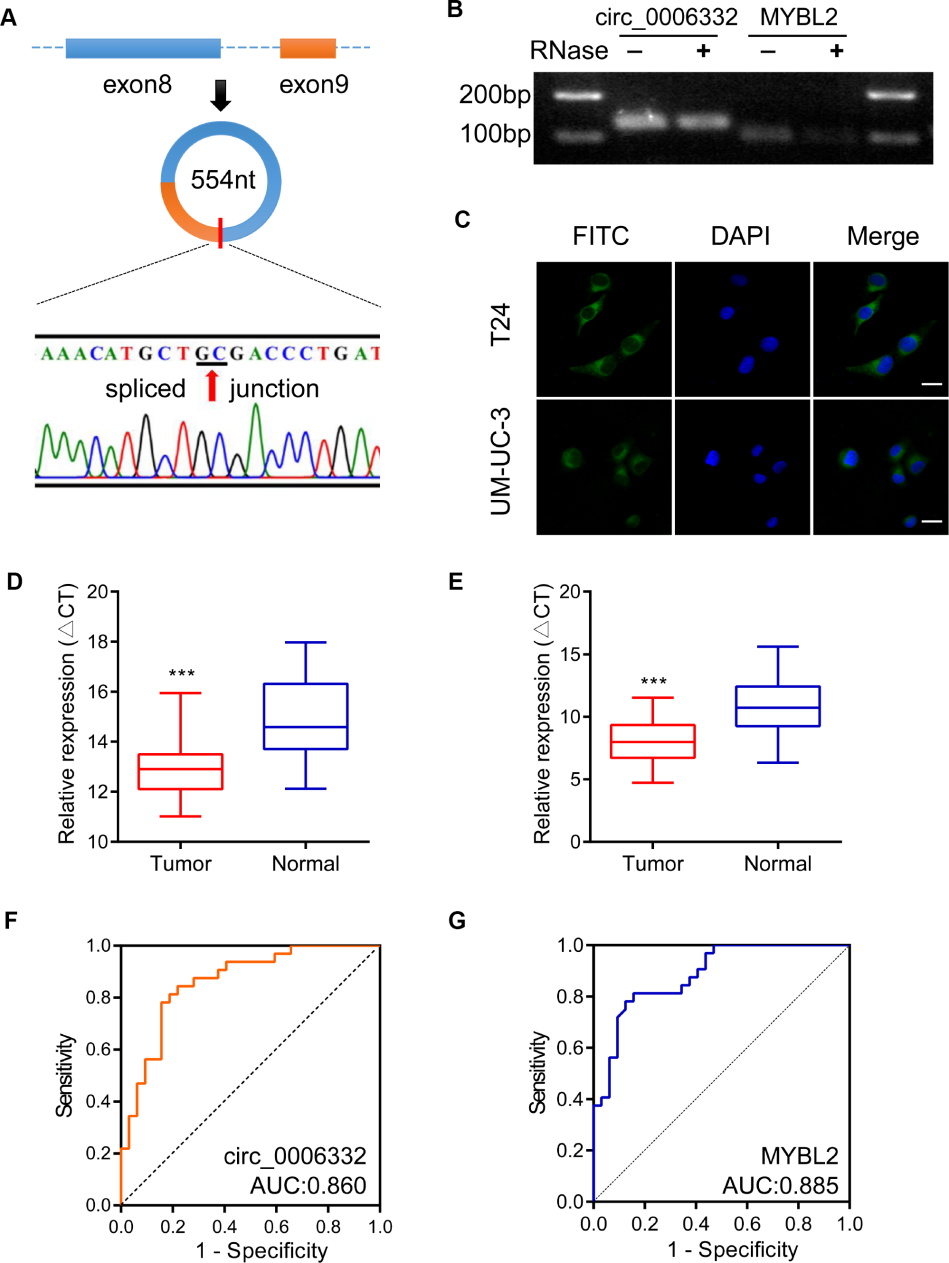
**Characteristics and clinical significance of circ_0006332.** (**A**) The diagram shows the structure and the splice junction of circ_0006332. Direct Sanger sequencing data shows that circ_0006332 is spliced out at the GC junction between exons 8 and 9 of the MYBL2 precursor mRNA transcript. (**B**) Agarose gel electrophoresis shows PCR products obtained from circRNA and linear mRNA samples after digestion with Ribonuclease R. The results show that the PCR primer was specific for the circRNAs and circRNAs were resistant to digestion by the exonuclease. (**C**) Representative FISH images show that circ_0006332 is located in the cytoplasm of bladder cancer cells. Scale bar: 50 μm. (**D**) Quantitative RT-PCR analysis of circ_0006332 expression in 32 paired specimens of bladder cancer and adjacent normal bladder tissues is shown. The expression is represented by the delta cycle threshold (ΔCT). Lower ΔCT value corresponds to high expression of circ_0006332. (**E**) Quantitative RT-PCR analysis shows MYBL2 mRNA expression in 32 coupled specimens of bladder cancer and adjacent normal bladder tissues. (**F**) Receiver operating characteristic (ROC) curve analysis of circ_0006332 expression in the clinical diagnosis of bladder cancer. The area under the curve (AUC) value: 0.860, sensitivity: 80.2%, specificity: 86.0%. (**G**) ROC curve analysis of MYBL2 expression in the clinical diagnosis of bladder cancer. The AUC value: 0.885, sensitivity: 81.3%, specificity: 84.6%. Note: Data were represented as mean ± SD; ***P < 0.001.

Circ_0006332 is 554 nucleotides in length and is generated by splicing between exons 8 and 9 of the *MYBL2* transcript (Figure 2A). Agarose gel electrophoresis showed that circ_0006332 was resistant to exonuclease, whereas, the linear *MYBL2* mRNA was sensitive and digested by the exonuclease (Figure 2B). Fluorescence *in situ* hybridization (FISH) showed that circ_0006332 is localized in the cytoplasm of T24 and UM-UC-3 cells (Figure 2C). QRT-PCR analysis of 32 bladder cancer and adjacent normal tissue samples showed that circ_0006332 was significantly upregulated in bladder cancer tissues (Figure 2D). Similarly, MYBL2 mRNA levels were significantly higher in the bladder cancer tissues compared with the adjacent normal bladder tissues (Figure 2E). The area under the curve (AUC) values for circ_0006332 and MYBL2 were 0.860 and 0.885, respectively (Figure 2F and 2G), thereby demonstrating their potential as early diagnostic markers for bladder cancer. The expression of circ_0006332 correlated with tumor-node-metastasis (TNM) stage and muscular invasion (Table 1).

**Table 1:** Correlation of circ_0006332 expression with clinic-pathologic characteristics of bladder patients.

**Characteristics**	**No of Cases**	**Mean±SD**	***P* value**
Age(years)			
≥60	25	3.82±5.32	0.395
< 60	7	8.02±9.72	
Gender			
Male	27	5.24±6.81	0.188
Female	5	2.72±2.05	
Grade			
G1	9	2.05±1.07	0.036
G2	—	—	
G3	23	5.42±7.09	
Diameter(cm)			
≥5	5	4.76±2.84	0.531
< 5	27	3.59±2.41	
TNM stage			
1	13	1.99±1.40	0.082
2	12	5.34±7.72	
3	5	8.88±7.93	
4	2	0.84±0.75	
Lymphatic metastasis			
N1-2	2	0.84±0.75	0.401
N0	30	4.71±6.32	
Invasion depth			
NMIBC	13	2.02±1.35	0.031
MIBC	19	6.38±7.73	

TNM, tumor-node-metastasis; N1-2, 1-2 lymphatic node; N0, 0 lymphatic node; NMIBC, non-muscular invasion bladder cancer; MIBC, muscular invasive bladder cancer.

### Knockdown of circ_0006332 inhibits proliferation and invasion of bladder cancer

**Figure 3:**
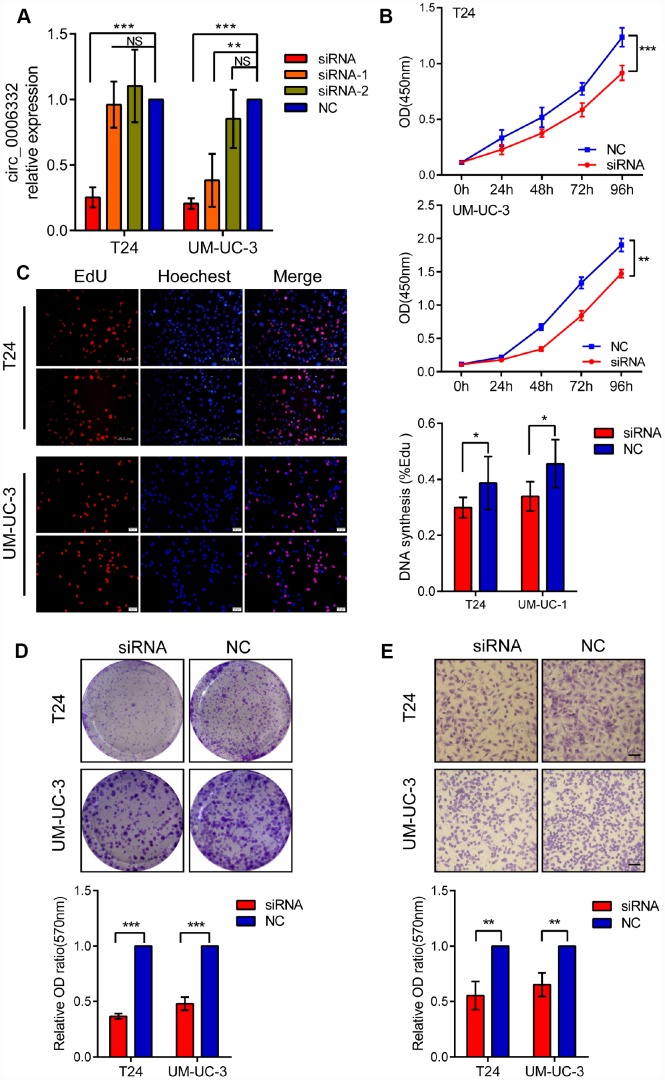
**Knockdown of circ_0006332 decreases cell proliferation, colony formation, and the invasiveness of bladder cancer cells.** (**A**) The qRT-PCR analysis shows that bladder cancer cells transfected with siRNA against circ_0006332 significantly reduce the expression of circ_0006332 compared with the controls. Note: siRNA, short interfering RNA against circ_0006332; NC, negative control; siRNA-1, siRNA containing 12 nucleotides from the 5′ end of the siRNA against circ_0006332 and 7 nucleotides from the negative control sequence; siRNA-2, siRNA containing 7 nucleotides from the 3′ end of the siRNA against circ_0006332 and 12 nucleotides from the negative control sequence. (**B**) CCK-8 assay shows decreased viability of circ_006332-knockdown T24 and UM-UC cells. (**C**) EdU detection assay shows that circ_006332 knockdown decreases the proportion of cells undergoing DNA synthesis compared with the controls. (**D**) The graph shows total number of colonies in control and circ_006332-knockdown bladder cancer cells. As shown, circ_006332 knockdown decreases the total number of colonies and the size of the colonies from the bladder cancer cells. (**E**) Transwell assay results show the total number of cells that invaded the bottom chamber in control and circ_006332-knockdown bladder cancer cell lines in 24 h. As shown, circ_006332 knockdown decreases the invasiveness of bladder cancer cells. Note: All experiments were repeated three times. The data are represented as mean ± SD; *P < 0.05, **P < 0.01, ***P < 0.001.

As shown in Figure 3A and Supplementary Figure 1A, we inhibited the expression of circ_0006332 using circ_0006332-specific siRNA in T24 and UM-UC-3 cells. Cell Counting Kit-8 (CCK-8) assays showed that the knockdown of circ_0006332 significantly decreased the viability of T24 and UM-UC-3 cells compared with the controls (Figure 3B). EdU (5-ethynyl-2′-deoxyuridine) assays showed that the knockdown of circ_0006332 significantly decreased the number of cycling cells or cells undergoing DNA synthesis (Figure 3C). The colony size and numbers of the circ_0006332-knockdown T24 and UM-UC-3 cells was significantly lower than the negative control groups (Figure 3D). Transwell cell invasion assays showed that the knockdown of circ_0006332 significantly decreased the cell invasiveness of the T24 and UM-UC-3 cells (Figure 3E). These results suggest that circ_0006332 promotes cell proliferation and invasiveness of bladder cancer cells.

### Circ_0006332 increases MYBL2 expression by sponging miR-143

**Figure 4:**
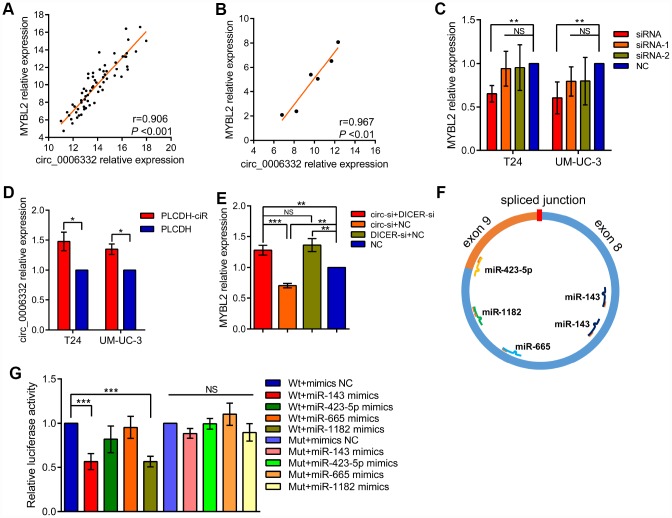
**Circ_0006332 increases MYBL2 expression by sponging miR-143.** (**A**–**B**) Correlation analysis shows that circ_0006332 levels correlate with MYBL2 expression in bladder cancer tissues and cell lines including 5637, T24, J82, UM-UC-3, TSCCUP and SV-HUC-1. (**C**) QRT-PCR analysis shows MYBL2 mRNA levels in bladder cell lines transfected with siRNA, siRNA-1, siRNA-2 and negative control against circ_0006332. As shown, MYBL2 expression is significantly decreased in circ_0006332 knockdown bladder cancer cells, but is not downregulated in cells transfected with siRNA-1 and siRNA-2. (**D**) QRT-PCR analysis shows MYBL2 mRNA levels in control and circ_0006332 overexpressing bladder cancer cells. Bladder cancer cells were transfected with PLCDH-ciR, the cloning vector with circ_0006332 or control vector, PLCDH. As shown MYBL2 mRNA levels are higher in cells overexpressing circ_0006332 compared with controls. (**E**) QRT-PCR analysis shows MYBL2 mRNA levels in bladder cancer cells transfected with negative control and siRNA against Dicer. As shown, Dicer knockdown cells eliminate the effects of circ_0006332 on MYBL2 expression. DICER-si, siRNA of DICER; circ-si, siRNA of circ_0006332 (**F**) MiRwalk and CircInteractome predict 4 miRNAs most likely bind to circ_0006332. As shown, circ_0006332 potentially bind to miR-143, miR-423-5p, miR-665, miR-1182 at position of 180-186 and 222-227, 458-464, 343-349, 382-395 respectively. (**G**) Results of the dual luciferase reporter assays confirm that miR-143 binds to wild type circ_0006332 (Wt) but do not bind to mutant circ_0006332 (Mut). Note: All experiments were repeated thrice and data were represented as mean ± SD; NS, no significance; *P < 0.05, **P < 0.01, ***P < 0.001.

**Figure 5:**
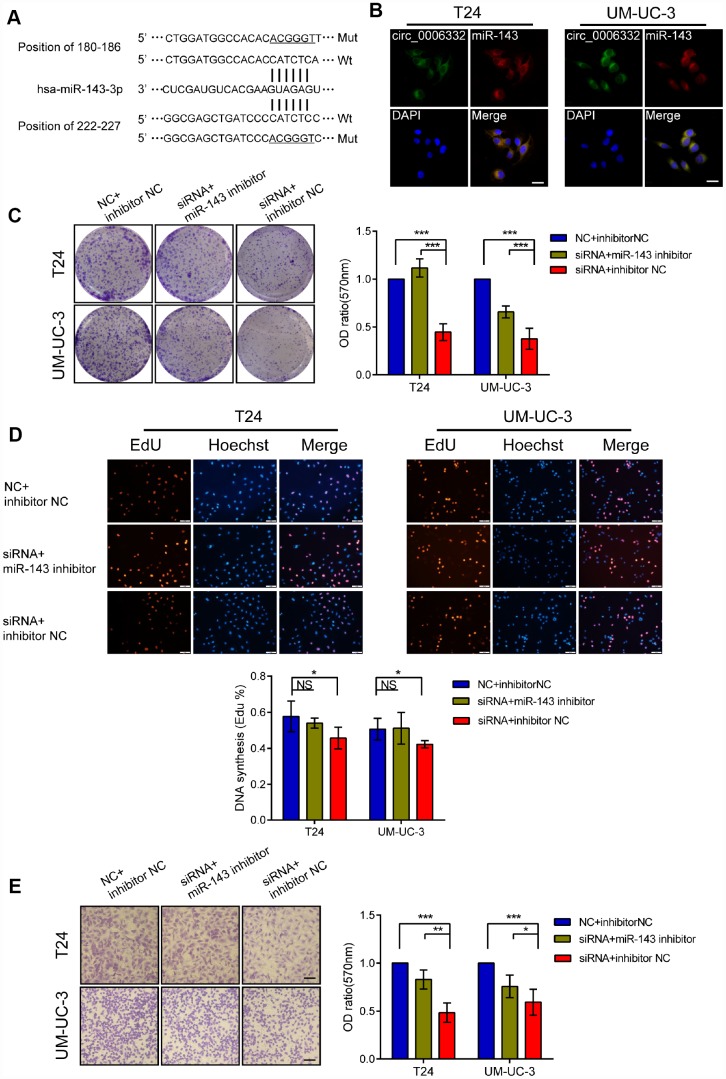
**Circ_0006332 promotes bladder cancer cell proliferation, colony formation, and invasiveness by binding and inhibiting miR-143.** (**A**) Predicted wild-type (Wt) and mutant (Mut) miR-143 binding sites in the sequence of circ_0006332 is shown. (**B**) Representative images of the FISH analysis show co-localization of circ_0006332 with miR-143 in the cytoplasm of bladder cancer cells. Scale bar: 50 μm. (**C**) Colony formation assay results show that bladder cancer cells transfected with miR-143 mimics form smaller and fewer colonies compared with the controls. (**D**) EdU detection assays shows that bladder cancer cells transfected with miR-143 mimics decreases the proportion of cells undergoing DNA synthesis compared with the controls. (**E**) Representative images of the Transwell assay show decreased invasiveness of the bladder cancer cells transfected with miR-143 mimics compared with the controls. Scale bar: 50 μm. Note: All experiments were repeated thrice; data is represented as mean ± SD; NS, no significance; *P < 0.05, **P < 0.01, ***P < 0.001.

QRT-PCR showed that the expression of circ_0006332 positively correlated with the expression of MYBL2 in both bladder cancer tissues and 6 cell lines including 5637, T24, J82, UM-UC-3, TSCCUP and SV-HUC-1 (Figure 4A and 4B). Circ_0006332 knockdown by siRNA significantly decreased the expression of MYBL2 (Figure 4C and Supplementary Figure 1B), whereas, overexpression of circ_0006332 (Supplementary Figure 1C) significantly increased the expression of MYBL2 in the bladder cancer cell lines T24 and UM-UC-3 (Figure 4D). Meanwhile, siRNA-1 and siRNA-2 against circ_0006332 did not significantly change the expression of MYBL2 (Figure 4C). DICER knockdown (Supplementary Figure 1D) significantly decreased the ability of circ_0006332 to regulate MYBL2 expression (Figure 4E). This suggests that circ_0006332 promotes MYBL2 expression by sponging regulatory miRNAs. We found that circ_0006332 and MYBL2 have common potential binding sites to miR-143, miR-423-5p, miR-665 and miR-1182 (Figure 4F and Supplementary Table 2). Luciferase reporter assay results showed that miR-143 specifically binds to circ_0006332 (Figure 4G and Supplementary Figure 1E). Circ_0006332 has two potential binding sites for miR-143 basing on prediction of miRwalk and CircInteractome (Figure 5A), and it co-localizes with miR-143 in the cytoplasm of bladder cancer cells (Figure 5B). Knockdown of circ_0006332 significantly decreases miR-143 expression (Supplementary Figure 1F). Furthermore, co-transfection of the circ_0006332 siRNA and the miR-143 inhibitor increased the number and size of colonies (Figure 5C), DNA synthesis (Figure 5D), and cell invasiveness (Figure 5E) of bladder cancer cell lines compared with the cells that were transfected with circ_0006332 siRNA only. This suggests that circ_0006332 increases RNA expression of MYBL2 by sponging miR-143 in the bladder cancer cells.

### MiR-143 decreases MYBL2 expression

**Figure 6:**
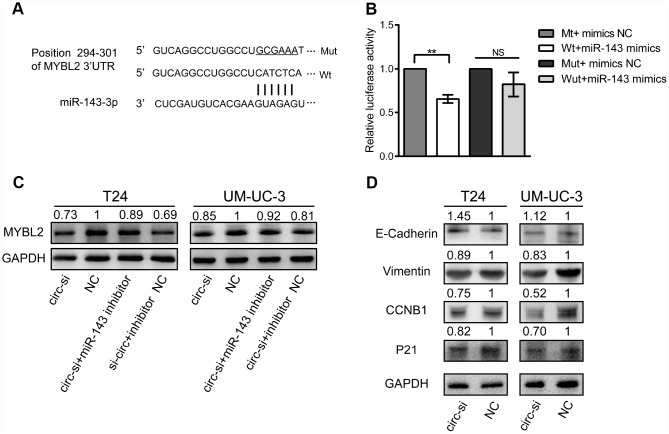
**MiR-143 downregulates MYBL2, and circ_0006332 promotes EMT and cell cycle progression of bladder cancer cells.** (**A**) Diagram shows potential miR-143 binding sites in the 3′ UTR region of the MYBL2 transcript. (**B**) Results of dual luciferase reporter assay show that miR-143 binds to the wild type MYBL2 3′UTR but can’t bind to mutant MYBL2 3′UTR. (**C**) Western blot results show MYBL2 protein levels in bladder cancer cells transfected with siRNA against circ_0006332 alone or siRNA against circ_0006332 alone + miR-143 inhibitor. As shown, miR-143 inhibitor increases MYBL2 protein levels. (**D**) Western blot analysis shows that knockdown of circ_0006332 increases E-cadherin and decreases Vimentin, CCNB1, P21 protein levels compared with the controls. Note: All experiments were repeated thrice; data are represented as mean ± SD; NS, no significance; **P < 0.01.

We identified a potential binding site of miR-143 in the 3′ untranslated region (UTR) of MYBL2 (Figure 6A). We performed luciferase reporter assays to confirm that miR-143 regulates MYBL2 expression. We observed that relative luciferase activity was significantly decreased in T24 cells co-transfected with the pmirGLO vector and mimics of miR-143 (Figure 6B). Western blot analysis showed that MYBL2 protein levels were significantly decreased in T24 cells transfected with circ_0006332 siRNA (Figure 6C). Conversely, inhibition of miR-143 increased MYBL2 protein expression despite circ_0006332 knockdown by siRNA (Figure 6C). Besides, the knockdown of circ_0006332 increased the expression of E-cadherin and decreased the expression of Vimentin, CCNB1 and P21 (Figure 6D). This suggests that circ_0006332 regulates epithelial-mesenchymal transition (EMT) and cancer progression of bladder cancer cells. These data further confirm that MYBL2 expression is regulated by circ_0006332 and miR-143 in the bladder cancer cells.

### Circ_0006332 promotes bladder cancer growth

**Figure 7:**
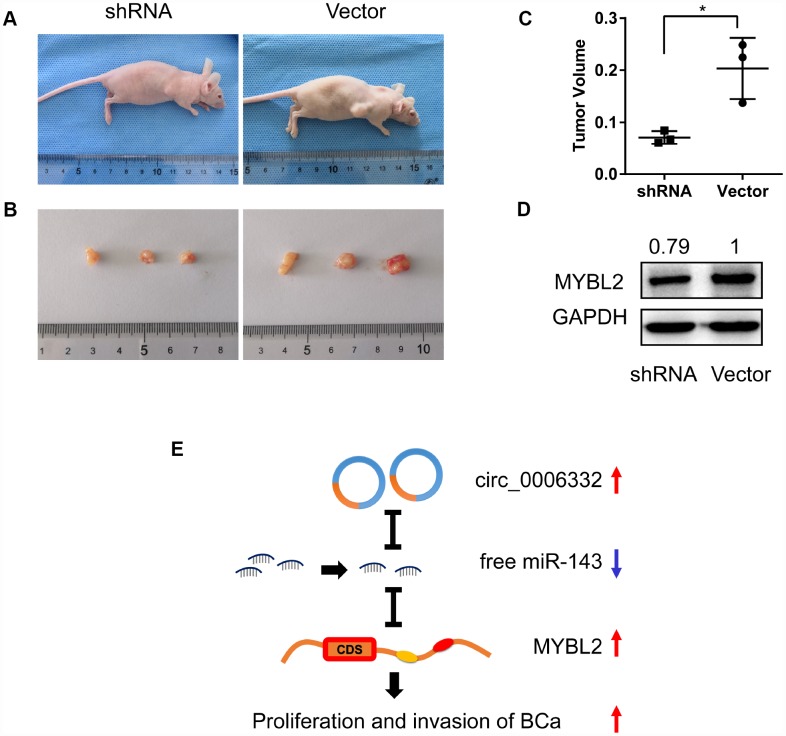
**Circ_0006332 promotes growth of bladder cancer xenograft *in vivo*.** (**A**) *In vivo* experiment strategy involved subcutaneously injecting nude mice with T24 bladder cancer cells transfected with shRNA against circ_0006332 or the empty vector. (**B**) Xenograft tumor size at day 30 in the circ_0006332 shRNA and empty vector groups is shown. (**C**) Xenograft tumor volumes at day 30 in the circ_0006332 shRNA and empty vector groups are shown. (**D**) Western blot analysis shows significantly lower MYBL2 protein levels in the circ_0006332 shRNA group tumors compared with the empty vector group tumors. (**E**) Upregulated circ_0006332 sponges and decreases free miR-143 levels, which increases MYBL2 expression and leads to bladder cancer (BCa) tumorigenesis. Note: Data are represented as mean ± SD; *P < 0.05.

Next, we performed *in vivo* experiments in nude mice to understand the role of circ_0006332 in bladder cancer. We subcutaneously injected bladder cancer cells transfected with control or circ_006332 shRNA. At day 30, we observed a 45% reduction in tumor volume in mice xenografted with T24 cells with circ_006332 shRNA compared with the vector control group (Figure 7A–7C). We further observed significantly lower MYBL2 protein levels in xenograft tumors derived from circ_0006332 knockdown bladder cancer cells compared with the controls (Figure 7D). These data suggest that circ_0006332 regulates growth and progression of bladder cancer by upregulating MYBL2 by sponging miR-143.

## DISCUSSION

In our study, RNA-seq analysis shows that circ_0006332 is a novel circRNA generated by splicing within the *MYBL2* transcript. Circ_0006332 is overexpressed in bladder cancer tissues and correlates with TNM stage and muscular invasiveness. Circ_0006332 knockdown inhibits *in vitro* proliferation and invasiveness of bladder cancer cell lines, and suppresses *in vivo* xenograft tumor growth in nude mice. Our study suggests that circ_0006332 acts as a sponge for miR-143 and promotes high expression of MYBL2 that drives bladder tumorigenesis. Thus, our study suggests that circ_ 0006332 is a potential diagnostic biomarker of bladder cancer.

Sanger forward sequencing and FISH analysis [13] shows that circ_0006332 has a special ring structure and is generated from the sequence of the splice junction between exons 8 and 9 of the *MYBL2* transcript. Since most circRNAs are composed of sequences from the coding exons, the linear mRNAs can prevent their detection by interference. Therefore, we digested linear mRNAs with Ribonuclease R, a nucleic acid exonuclease, to accurately characterize the circular RNAs [14]. Since circRNAs do not have a polyA tail, we used random primers instead of oligo dT primers to amplify them by reverse transcription [15]. Conversely, oligo dT primers amplify only the linear mRNAs. We also used convergent primers to amplify and confirm that circ_0006332 is derived from *MYBL2* transcript [16]. We further used Ribonuclease R digestion of RNA samples followed by reverse transcription to ensure accurate and credible characterization of the circRNAs in this study.

CircRNAs have emerged as important biomarkers for many tumors. For example, low expression of hsa_circ_002059 correlates with overexpression of carcinoembryonic antigen (CEA) and distant metastasis in gastric cancer [17]. CircHIPK3 is significantly downregulated in bladder cancer tissues, and negatively correlates with cancer grade, tumor invasion and lymph node metastasis [18]. These results suggest the potential of circRNAs in the clinical diagnosis and prognosis of tumors. The AUC value for circ_0006332 is 0.885, which suggests that it could be a highly sensitive and specific biomarker for the diagnosis of bladder cancer. High expression of circ_0006332 significantly correlates with clinical TNM stage and muscular invasion. Circular RNAs are more stable than linear RNAs and may be more accurate biomarkers because processing of blood and urinary samples usually takes time [19]. Therefore, our results show that circ_0006332 may be a useful early diagnostic biomarker for bladder cancer.

MYBL2 belongs to the MYB transcription factor family [20], and promotes cell proliferation by helping cells to overcome the G2 phase checkpoint [21]. MYBL2 is overexpressed in hepatic cancer [22], breast cancer [23] and bladder cancer [24]. In advanced prostate cancer, MYBL2 expression positively correlates with metastasis [25]. Our results show that MYBL2 is significantly overexpressed in bladder cancer cells and tissues. Our data also shows that circ_0006332 promotes bladder cancer cell proliferation and invasion, and regulates MYBL2 expression. When circ_0006332 expression is high, MYBL2 is overexpressed, whereas knockdown of circ_0006332 downregulates MYBL2 expression. Besides, we used two specific siRNAs, siRNA-1 and siRNA-2, to eliminate the off-target effects of circ_0006332 siRNAs. MYBL2 expression was unchanged in bladder cancer cells after transfection with siRNA-1 and siRNA-2. Moreover, circ_0006332 levels positively correlate with MYBL2 expression in the bladder cancer tissues and cells. These data confirm that circ_0006332 promotes bladder cancer growth and progression by increasing MYBL2 expression.

Most circRNAs are enriched with miRNA binding sites, and can sponge miRNAs to relieve their suppression of target mRNAs [26]. DICER is essential for the maturation of miRNAs. It cleaves short hairpin pre-miRNAs into mature miRNAs that are 21 to 23 nucleotides in length and have 3′ overhangs of two nucleotides [27]. We observed that inhibition of DICER altered the regulation of MYBL2 by circ_0006332. We predicted that circ_0006332 acted as a sponge for miRNAs because it was located in the cytoplasm of bladder cancer cells. We used multiple binding site prediction software to narrow down on a limited number of miRNAs that could potentially bind to circ_0006332 [28–30]. We confirmed that circ_0006332 specifically binds miR-143 in the cytoplasm using co-localization studies and luciferase reporter assays. We also showed that miR-143 is downregulated in circ_0006332 knockdown T24 and UM-UC-3 cells as shown previously [31, 32]. Most likely, we obtained this result because we detected total amounts of miRNAs (both bound and free miRNAs) and are therefore examining dynamic changes in the levels of total miRNAs.

In most tumors, miR-143 acts as a tumor suppressor [33, 34]. In colorectal cancer cells, miR-143 inhibits cell proliferation by decreasing MMP7 expression [35]. Furthermore, miR-143 inhibits cell proliferation, invasion, and epithelial-mesenchymal transition in esophageal squamous cell cancer [36]. MiR-143 also reduces bladder cancer cell growth and migration by targeting cyclooxygenase-2 [37]. We used dual luciferase reporter assays to demonstrate that miR-143 inhibits MYBL2 expression by binding to its 3′ UTR. These results suggest that circ_0006332 acts as a sponge for miR-143, which inhibits proliferation and invasion of bladder cancer cells by targeting MYBL2 (Figure 7E). MYBL2 has previously been reported to promote EMT and cell cycle progression [38]. In our study, we demonstrate that knockdown of circ_0006332 increases E-cadherin expression and decreases Vimentin, P21 and CCNB1 expression. This suggests that circ_0006332 promotes EMT and cell cycle progression of bladder cancer cells.

In conclusion, our study demonstrates that circ_0006332 increases MYBL2 expression by acting as a sponge for miR-143, and promotes the proliferation and invasion of bladder cancer. We postulate that circ_0006332 is a potential diagnostic biomarker of bladder cancer.

## MATERIALS AND METHODS

### Population

Thirty-two patients diagnosed with bladder cancer were included in this study. Fresh bladder cancer and adjacent normal bladder tissue samples were collected between December 2016 and July 2017 during radical surgery of these patients at the Fourth Affiliated Hospital of China Medical University. The samples were frozen in liquid nitrogen for 1 h and stored at −80°C. None of the enrolled patients received chemotherapy, radiotherapy or targeted therapy before radical surgery. The study protocol was approved by the Institutional Review Board of China Medical University (Shenyang, China). Informed consent was obtained from all patients involved in this study.

### Sequencing analysis

We performed whole transcriptome sequencing using HiSeq X instrument (illumina, San Diego, CA, USA) on four out of the 32 paired bladder cancer and adjacent normal bladder tissues. We identified differentially expressed transcripts between tumor and normal tissue samples by using the criterion of |log2 (fold-change)|>1 and a P value < 0.05. The CIRCexplorer (http://yanglab.github.io/CIRCexplorer/) and circBase (http://www.circbase.org/) databases were used to predict the spliced circRNAs and to acquire the circRNA sequences.

### Cell culture

Human bladder cancer cell lines 5637, T24, J82, UM-UC-3, TSCCUP and SV-HUC-1 were purchased from the Cell Bank of Type Culture Collection (Shanghai, China). The cell lines were maintained in MEM (HyClone, Logan, UT, USA) or RPMI 1640 (HyClone) media containing 10% fetal bovine serum (HyClone) in a humidified incubator at 37°C and 5% CO_2_.

### Fluorescence *in situ* hybridization

Specific probes of circ_0006332 and miR-143 were used for *in situ* hybridization. The circ_0006332 probe sequence was 5′-CAAGCATCAGGGTCGCAGCAT GTTTCTGGT-3′ and was labeled with fluorescein isothiocyante (FITC). The miR-143 probe sequence was 5′-GAGCTACAGTGCTTCATCTCA-3′ and was labeled with Cyanine3 (Cy3). We used 4,6-diamidino-2-phenylindole (DAPI) for staining the nuclei. All procedures were conducted according to the manufacturer’s instructions (Servicebio, Wuhan, China). The images were acquired using the NIKON ECLIPSE TI-SR microscope system (Nikon, Tokyo, Japan).

### Total RNA extraction and qRT-PCR

Trizol (Invitrogen, Carlsbad, MA, USA) was used to extract total RNA from the patient tissue samples according to the manufacturer’s instructions. To estimate the levels of specific mRNAs and circRNAs, cDNA synthesis was performed by reverse transcription using a High-Capacity cDNA Reverse Transcription Kit (Thermo Fisher, MA, USA). Then, equal amounts of cDNA were used for qRT-PCR using the TB Green Premix Ex Taq II kit (Takara, Dalian, China). To quantify miRNA levels, cDNA synthesis was performed by reverse transcription using the miRcute Plus miRNA First-Strand cDNA Kit (Tiangen, Beijing, China). Then, qRT-PCR was performed using the SYBR Green miRcute Plus miRNA qPCR Kit (Tiangen). All reactions were performed in triplicate. The cycling conditions were according to the manufacturer’s instructions. All primers are synthesized by Shanghai Shenggong Company (Shanghai, China) and listed in the Supplementary Table 3.

### Exonuclease protection assay

Exonuclease protection assay was performed using Ribonuclease R (Epicentre, San Diego, CA, USA) from *E. coli* according to the manufacturer’s instructions. Briefly, circRNA and its parent mRNA were both digested by Ribonuclease R. Then, random primers (Takara) were used to reverse transcribe the circRNAs, and then PCR-amplified using specific divergent primers (Sangon). Oligo dT primers (Takara) were used to reverse transcribe the mRNAs and then PCR amplified using specific convergent primers (Sangon). The PCR products were separated on a 2% agarose gel electrophoresis (Thermo Fisher) and quantified.

### Target miRNA prediction

TargetScan (http://www.targetscan.org/vert_72/), miRwalk (http://mirwalk.umm.uni-heidelberg.de/), and CircInteractome (https://circinteractome.nia.nih.gov/) databases were used to predict potential miRNA targets of circ_0006332 and MYBL2.

### Transfections and vector construction

The circ_0006332 siRNA, miRNA mimics and miRNA inhibitor were all synthesized by GenePharma (Suzhou, China). Furthermore, two specific siRNAs (GenePharma), siRNA-1 and siRNA-2, were synthesized that had similar structure and length as the circ_0006332 siRNA. The siRNA-1 had 12 conserved nucleotides as at the 5′ end of the circ_0006332 siRNA, whereas, siRNA-2 had 7 conserved nucleotides as at the 3′ end of the circ_0006332 siRNA. The remaining sequences of siRNA-1 and siRNA-2 were from the negative control. The lentiviral vector containing circ_0006332 shRNA was constructed by Hanbio (Shanghai, China). A 606-bp cDNA fragment that contained the sequence of circ_0006332 was cloned into the PLCDH vector (GenePharma) to overexpress circ_0006332. The siRNAs, miRNA mimics, miRNA inhibitor, and the PLCDH vector transfections were performed according to the manufacturer’s instructions using the X-tremeGENE siRNA Transfection Reagent (Roche, Basel, Switzerland). The lentiviral transfections were performed according to the manufacturer’s instructions. The sequences of siRNAs, miRNA mimics, and miRNA inhibitor are listed in the Supplementary Tables 3.

### Dual Luciferase reporter assay

The mutant and the wild-type circ_0006332 sequences were synthesized and cloned into the pmirGLO vector (GenePharma). Moreover, a portion of the 3′UTR region of MYBL2 that contains the wild-type or the mutated miRNA binding sites were synthesized and cloned into the pmirGLO vector (GenePharma). Six nucleotides were mutated to generate the mutant circ_0006332 and MYBL2 sequences. Then, the T24 bladder cancer cells (5×10^4^) were seeded in 12-well plates and transiently transfected with the wild-type or mutant circ_0006332 or MYBL2 vectors. Furthermore, 75 nM miRNA mimics was co-transfected with 0.5 ug of vector into T24 cells. The transfected cells were harvested at 72 h after transfection. The Dual Luciferase Reporter Assay System (Promega, Madison, WI, USA) was used to detect luciferase activity.

### CCK-8 cell proliferation assay

The transfected cells were seeded into 96-well plates at a density of 2000 cells per well. Then, cell viability was measured at 0, 24, 48, 72 and 96 h using the Cell Counting Kit-8 (CCK-8) system (Dojindo, Tokyo, Japan), according to the manufacturer’s instructions. Briefly, cells were incubated at 37°C for 90 min in the dark after adding 10 μl of CCK-8 solution into each well at the appropriate time points. The absorbance was measured at 450 nm using a microplate reader (Bio-Rad, Hercules, CA, USA)

### Colony formation assay

Transfected cells were seeded into 6-well plates at a density of 500 cells per well and cultured in RPMI 1640 (Hyclone) or DMEM medium (Hyclone) containing 10% fetal bovine serum (Hyclone). After 1 week, the cells were fixed with methanol and stained with 0.1% crystal violet. The number of colonies was counted and their images were captured under a light microscope. Then, the fixed cells were treated with 33% ethanoic acid to extract the crystal violet, the solution was transferred to 96-well plates, and the absorbance was measured at 570 nm using a microplate reader (Bio-Rad).

### EdU assay

The transfected cells were seeded into 12-well plates at a density of 10000 cells per well, cultured with 20 μM EdU for 120 min, and then stained with the Fluor555 Click-iT EdU kit (KeyGEN, Nanjing, China). The images were acquired using the Olympus microscope (Olympus, Tokyo, Japan).

### Transwell cell invasion assays

For invasion assays, 4 × 10^4^ transfected cells were suspended in 100 μl of serum-free RPMI medium and seeded into the upper chambers of each Costar Transwell (8 μm pore size) that were coated with matrigel (BD Biosciences, San Jose, CA, USA). The bottom chamber contained RPMI medium supplemented with 10% fetal bovine serum as a chemoattractant. The cells were incubated at 37°C with 5% CO_2_ for 24 h. Then, the cells in the lower chamber were fixed with methanol, stained with 0.1% crystal violet, and photographed using an Olympus light microscope at ×100 magnification. Then, crystal violet dye was extracted from the stained cells with 33% ethanoic acid. The solution was transferred into 96-well plates, and the absorbance of each well was measured using a microplate reader (Bio-Rad) at 570 nm.

### Western blot

Cells were lysed with RIPA buffer (Beyotime, Beijing, China) and the extracted protein was quantified using bicinchoninic acid (Beyotime, Beijing, China). Protein samples (15 μg total protein per lane) were resolved by 10% SDS-PAGE and transferred onto polyvinylidene fluoride membranes (Millipore, Bedford, MA, USA). After blocking, the membranes were incubated with high-affinity anti-MYBL2 (1:500, Santa Cruz Biotechnology, Santa Cruz, CA, USA), anti-E-cadherin (1:1000, Abcam, Cambridge, MA, USA), anti-Vimentin (1:1000, Cell Signaling Technology, Danvers, MA, USA), anti-CCNB1 (1:1000, Cell Signaling Technology), anti-P21 (1:1000, Cell Signaling Technology) and anti-GAPDH (1:2000, Cell Signaling Technology) antibodies, overnight at 4°C. Then, after washing, the membranes were incubated with HRP-conjugated secondary monoclonal antibody (1:5000, Cell Signaling Technology) at room temperature for 1 hour. The blots were developed using a chemiluminescence system (Bio-Rad) and the protein bands were quantified.

### Xenograft tumors in nude mice

We purchased 5-week old athymic female BALB/C nude mice (18–22 g and 3 mice per group) from Charles River (Beijing, China). T24 cells were stably transfected with control or circ_0006332 shRNA. Then, 1×10^7^ cells were mixed in 100 μl matrigel (BD Biosciences) and 100 μl PBS and injected subcutaneously into the axilla of each mouse. The width (W) and the length (L) of the tumors were measured every week using calipers and the tumor volume (V) was calculated using the formula, V = (W^2^ × L)/2. The mice were euthanatized five weeks after injecting the cells. The subcutaneous tumors were surgically removed and weighed. The animal studies were performed in accordance with the institutional ethics guidelines for animal experiments and approved by the animal management committee of the China Medical University.

### Statistical analysis

All statistical analyses were performed using the SPSS 20.0 software (IBM, Armonk, NY, USA). Data were expressed as the means ± SD from at least three separate experiments. The differences between experimental groups were analyzed using the Student’s t test and the Kruskal–Wallis test. The correlation of expression between circ_0006332 and MYBL2 was analyzed using the Pearson correlation tests. The correlation between circ_0006332 expression and the clinical characteristics were analyzed using ANOVA. P values less than 0.05 were considered statistically significant.

## Supplementary Material

Supplementary Figure 1

Supplementary Tables
