# Supplementary material for: Circ_0006332 promotes growth and progression of bladder cancer by modulating MYBL2 expression via miR-143
Source: Aging (Albany NY). 2019 Nov 22;11(22):10626–43. doi: 10.18632/aging.102481 (PMC6914401; doi:10.18632/aging.102481)
Supplement: Supplementary Figure 1 [file aging-11-102481-s002..pdf]

SUPPLEMENTARY FIGURE

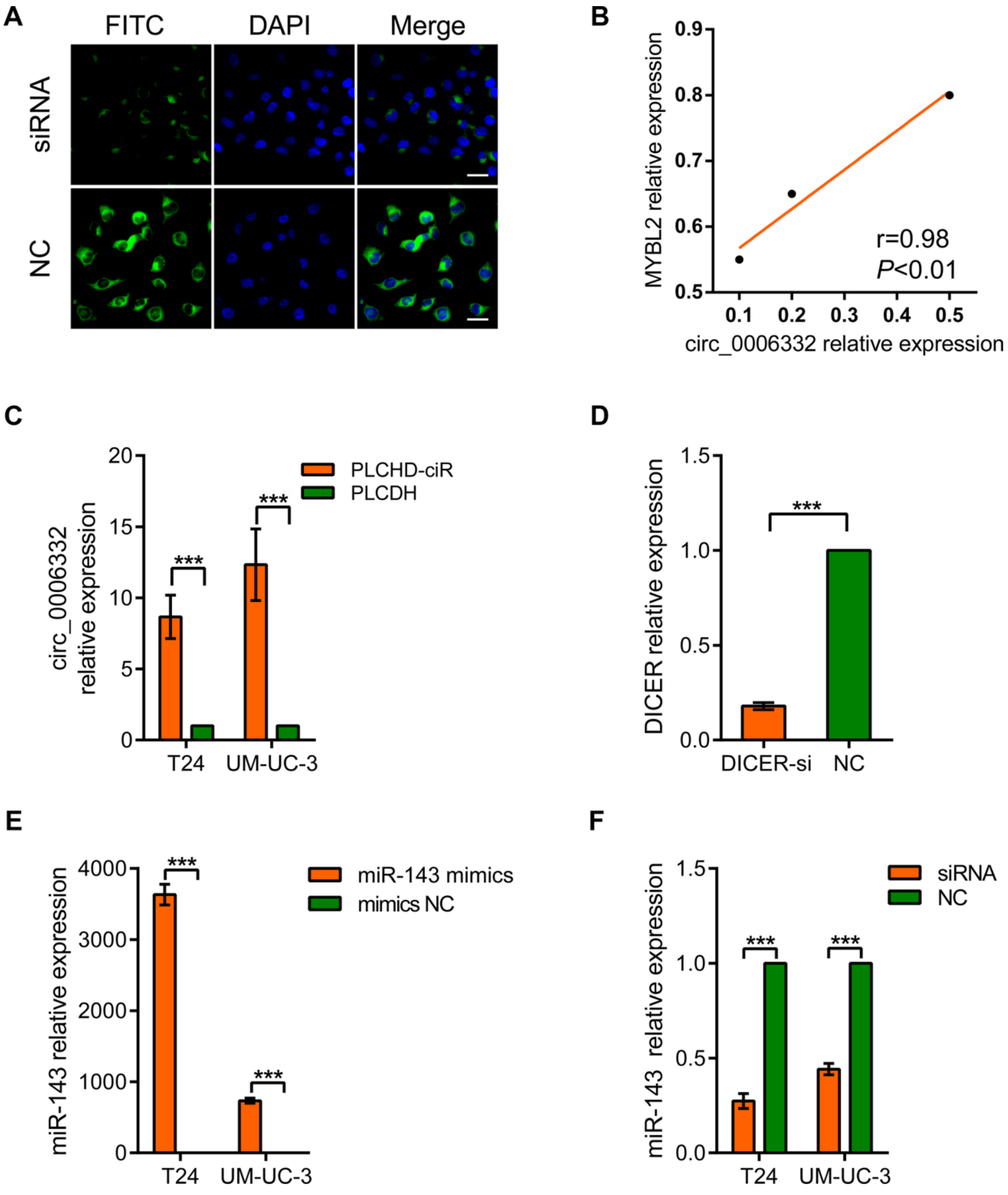

Supplementary Figure 1. Circ_0006332, DICER, and miR-143 levels in control and siRNA- or miRNA transfected bladder cancer cells. (**A**) Representative FISH images show siRNA against circ_0006332 decreases circ_0006332 expression in T24 cell. Scale bar: 50 μm. (**B**) Putative model shows probable mechanism of MYBL2 regulation by circ_0006332. QRT-PCR shows that circ_0006332 expression is inhibited by rates of 48%, 79% and 90% while MYBL2 expression is suppressed by rates of 19%, 25% and 36%. (**C**) Circ_0006332 levels in bladder cancer cells transfected with PLCDH-ciR and control vectors. (**D**) SiRNA against Dicer significantly reduces the expression of DICER compared with the controls in T24 cell. (**E**) MiR-143 mimics significantly increase the levels of miR-143 in the bladder cancer cells compared with the controls. (**F**) Circ_0006332 knockdown decreases miR-143 levels compared with the controls. Note: All experiments were repeated thrice; data are represented as mean ± SD; ***P < 0.001.
