# Supplementary material for: Circ_0006332 promotes growth and progression of bladder cancer by modulating MYBL2 expression via miR-143
Source: Aging (Albany NY). 2019 Nov 22;11(22):10626–43. doi: 10.18632/aging.102481 (PMC6914401; doi:10.18632/aging.102481)
Supplement: Supplementary Tables [file aging-11-102481-s001..pdf]

SUPPLEMENTARY TABLES

Supplementary Table 1. Information of selected circRNAs.

Gene name	Transcript Name	Biotype	Mapped circRNA ID	Exon/intron Count	log_2(Foldchange)	P value
FLNA	ENST00000610817	ciRNA	None	37	-3.59	0.004
KIAA1462	ENST00000375377	cirRNA	hsa_circ_0018069	3	-2.27	0.048
TBX20	ENST00000408931	cirRNA	None	6, 5, 4, 3, 2	-2.21	0.003
AKAP6	ENST00000280979	cirRNA	hsa_circ_0101667	3, 4, 5	-2.21	0.012
PGM5	ENST00000396396	cirRNA	hsa_circ_0138896	2, 3, 4, 5, 6, 7, 8, 9, 10	-1.99	0.020
TOX3	ENST00000219746	cirRNA	hsa_circ_0039351	3, 2	2.48	0.030
MOCOS	ENST00000261326	cirRNA	hsa_circ_0003552	2, 3, 4, 5	2.50	0.001
CCNB1	ENST00000256442	cirRNA	hsa_circ_0001495	6, 7	2.51	0.002
MYBL2	ENST00000396863	cirRNA	hsa_circ_0006332	7, 8	2.99	0.047
IGHG2	TCONS_00034765	ciRNA	None	2	4.07	0.004

ciRNA: circulated by intron, circRNA: circulated by exon

Supplementary Table 2. Wild and mutated type vector for circ_0006332.

Position of 180-186	5′	…CTGGATGGCCACACACGGGTTG…	3′	Mut
	5′	…CTGGATGGCCACACCATCTCAG…	3′	Wt
hsa-miR-143-3p	3′	CUCGAUGUCACGAAGUAGAGU	5′	
Position of 222-227	5′	…GGCGAGCTGATCCCACGGGTC…	3′	Mut
	5′	…GGCGAGCTGATCCCCATCTCC…	3′	Wt
hsa-miR-143-3p	3′	CUCGAUGUCACGAAGUAGAGU	5′	
Position of 458-464	5′	…CATTGGAGCTGGAGAAAAGGAAG…	3′	Mut
	5′	…CATTGGAGCTGGAGAGCCCCTCG…	3′	Wt
hsa-miR-423-5p	3′	UUUCAGAGCGAGAGACGGGGAGU	5′	
Position of 343-349	5′	…ACCAGTCTGTCCTATACAAA…	3′	Mut
	5′	…ACCAGTCTGTCCTTCCTGGA…	3′	Wt
hsa-miR-665	3′	UCCCCGGAGUCGGAGGACCA	5′	
Position of 382-395	5′	…AGAGCACACCTGTTATCGAGGAG…	3′	Mut
	5′	…AGAGCACACCTGTTAAGACCCTG…	3′	Wt
hsa-miR-1182	3′	CAGUGUAGGGAGGGUUCUGGGAG	5′	

Red nucleotides: predicted binding sites for miRNA and cir_0006332
Blue nucleotides: mutation nucleotides, Mut: mutated type, Wt: wild type.

Supplementary Table 3. Synthesized RNA sequence.

Dicer siRNA	GGUGGUCUUAGACAGGUAUTT
circ_0006332 siRNA	GAAACAUGCUGCGACCCUGTT
circ_0006332 siRNA-1	GAAACAUGCUGCCUCCGGATT
circ_0006332 siRNA-2	CUAACGAAUGCGGACCCUGTT
NC	UUCUCCGAACGUGUCACGUTT
miR-143 mimics	UGAGAUGAAGCACUGUAGCUC
miR-143 inhibitor	GAGCUACAGUGCUUCAUCUCA
miR-423-5p mimics	UGAGGGGCAGAGAGCGAGACUUU
miR-665 mimics	ACCAGGAGGCUGAGGCCCCU
miR-1182 mimics	GAGGGUCUUGGGAGGGAUGUGAC
mimics NC	UUCUCCGAACGUGUCACGUTT
inhibitor NC	CAGUACUUUUGUGUAGUACAA
circ_0006332 Forward Primer	ACCGGGACAAGACACCCC
circ_0006332 Reverse Primer	GCAGCTGCACTAGGCTGT
MYBL2 Forward Primer	GAGGGATAGCAAGTGCAAGGT
MYBL2 Reverse Primer	TTCCAGTCCTGCTGTCCAAA
Dicer Forward Primer	GTTTGAAAGCGTTGAGTGGTAT
Dicer Reverse Primer	GCCAGCTTCCTTTATCAATCTG
β-actin Forward Primer	GAGACCTTCAACACCCCAGCC
β-actin Reverse Primer	GGATCTTCATGAGGTAGTCAG
GAPDH Forward Primer	GGACCTGACCTGCCGTCTAG
GAPDH Reverse Primer	TAGCCCAGGATGCCCTTGAG
miR-143-5p Forward Primer	TGAGATGAAGCACTGTAGCTC
miR-370-3p Forward Primer	TTCCAGTCCTGCTGTCCAAA
miR-423-5p Forward Primer	TGAGGGGCAGAGCGAGACTTT
miR-665 Forward Primer	TCCCCGGAGTCGGAGGACCA
miR-1182 Forward Primer	CAGTGAGGGAGGGTTCTGGGAG
U6 Forward Primer	GCGCGTCGTGAAGCGTTC
Common Reverse Primer	GTGCAGGGTCCGAGGT
